# MicroRNAs in pulmonary arterial remodeling

**DOI:** 10.1007/s00018-013-1382-5

**Published:** 2013-06-06

**Authors:** Jennifer S. Grant, Kevin White, Margaret R. MacLean, Andrew H. Baker

**Affiliations:** Institute of Cardiovascular and Medical Sciences, British Heart Foundation Glasgow Cardiovascular Research Centre, University of Glasgow, 126 University Place, Glasgow, G12 8TA UK

**Keywords:** MicroRNAs, Remodeling, Smooth muscle cells, Endothelial cells, Pulmonary arterial hypertension

## Abstract

Pulmonary arterial remodeling is a presently irreversible pathologic hallmark of pulmonary arterial hypertension (PAH). This complex disease involves pathogenic dysregulation of all cell types within the small pulmonary arteries contributing to vascular remodeling leading to intimal lesions, resulting in elevated pulmonary vascular resistance and right heart dysfunction. Mutations within the bone morphogenetic protein receptor 2 gene, leading to dysregulated proliferation of pulmonary artery smooth muscle cells, have been identified as being responsible for heritable PAH. Indeed, the disease is characterized by excessive cellular proliferation and resistance to apoptosis of smooth muscle and endothelial cells. Significant gene dysregulation at the transcriptional and signaling level has been identified. MicroRNAs are small non-coding RNA molecules that negatively regulate gene expression and have the ability to target numerous genes, therefore potentially controlling a host of gene regulatory and signaling pathways. The major role of miRNAs in pulmonary arterial remodeling is still relatively unknown although research data is emerging apace. Modulation of miRNAs represents a possible therapeutic target for altering the remodeling phenotype in the pulmonary vasculature. This review will focus on the role of miRNAs in regulating smooth muscle and endothelial cell phenotypes and their influence on pulmonary remodeling in the setting of PAH.

## Introduction

Lung vasculopathy is an irreversible pathologic hallmark of the lung vascular disorder pulmonary arterial hypertension (PAH). PAH is an often fatal and increasingly prevalent disease that is manifested by a maladaptive elevation of pulmonary vascular resistance and pulmonary arterial pressure, consequently leading to right heart failure and eventual death. Clinically, the disease is defined as a mean pulmonary artery pressure of >25 mmHg at rest [1].

There are three main forms of PAH; idiopathic (IPAH), in which the cause is unknown, familial (FPAH), and PAH associated with other risk factors (APAH), such as HIV infection, collagen vascular diseases, and congenital heart disease [2]. The main genetic defect associated with PAH is a mutation in the gene encoding bone morphogenetic protein receptor 2 (BMPR2). Germline mutations in BMPR2 were originally identified in patients with FPAH [3, 4]. In these families, the disease segregates in an autosomal dominant fashion, with markedly reduced penetrance of approximately 20–30 % [5]. As such, many patients who carry the disease gene do not develop clinical PAH. In addition, up to 25 % of patients with apparently sporadic IPAH have been found to harbor similar mutations [6]. A proportion of these mutation carriers are examples of FPAH where the condition has not manifested in relatives due to low penetrance, while others are examples of de novo mutations. The low penetrance of the disease among BMPR2 mutation carriers suggests that other factors are important in the manifestation of clinical PAH and that a “second hit” in addition to a mutation in BMPR2 is required to establish PAH [7].

The incidence of PAH varies from 1.1, 2.0, and 2.4 per million of adult population per year in the UK and Ireland, USA, and France, respectively [8–10]. Recent studies show that females are more susceptible to developing PAH with a female-to-male ratio of 4.3:1 [11] in PAH and 4.1:1 in IPAH [12]. However, severity and survival is worse in males who have developed the disease than in females [13]. This clear difference between the genders is an intriguing phenomenon and much work is underway to identify the role of sex hormones such as estrogen on the development and maintenance of PAH.

**Fig. 1:**
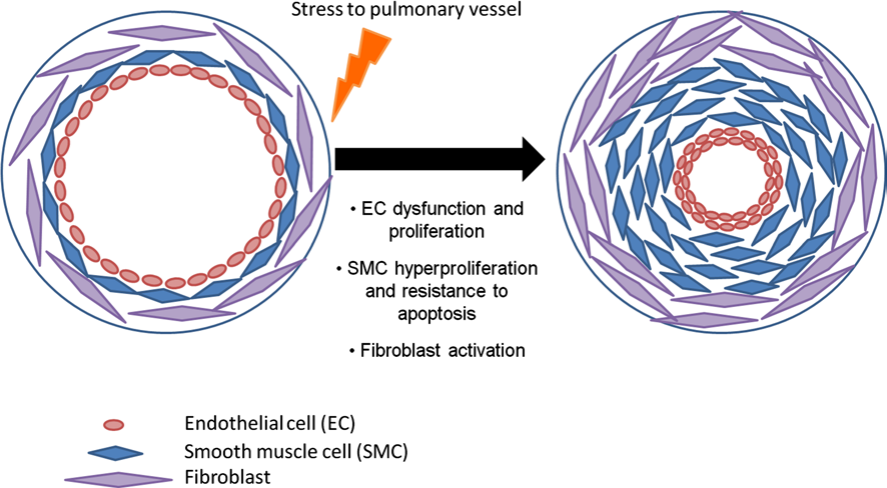
Pathogenesis of pulmonary arterial hypertension. Stress to the small pulmonary arteries results in endothelial dysregulation and proliferation in the intima, smooth muscle cell proliferation, and resistance to apoptosis within the medial layer, along with adventitial fibroblast activation. This culminates in vasoconstriction and remodeling of the pulmonary vessels, which can result in plexiform lesions in human PAH

Current treatments for PAH include endothelin-1 receptor antagonists, phosphodiesterase type 5 inhibitors, and administration of prostacyclins [14]. Although current therapies do indeed provide a survival benefit, mortality rates still remain high and the treatment does not prevent the aggressive progression of the disease. As a result, newer treatments are required to more effectively manage PAH and regulate the cellular components resulting in pulmonary remodeling. Contributing factors leading to remodeling include vessel injury, hypoxic exposure, and inflammation, resulting in severe remodeling of predominantly the small pulmonary vessels [15]. This remodeling process involves interaction between all cell types present in the distinct layers of the pulmonary arteries causing histological changes to the pulmonary vessel wall [16, 17] (Fig. 1).

The principal cell type within the adventitia layer of the vessel wall is the fibroblast, and adventitial thickening is observed due to hyperplasia of these cells [18, 19]. Activation of the fibroblasts results in a release of growth factors such as endothelin-1, serotonin, and platelet-derived growth factor (PDGF) [20] causing migration, proliferation, and contraction of fibroblasts and smooth muscle cells (SMC) [21]. An increase in reactive oxygen species (ROS) in the adventitia by NADPH oxidase can lead to activation of fibroblasts. From these activated adventitial fibroblasts, a subset population differentiate into myofibroblasts, characterized by α-SMA expression [22]. Myofibroblasts are mesenchymal cells that can be induced under the control of growth factors such as transforming growth factor (TGF-β1) [23]. These cells have the ability to migrate into the medial layer, increasing medial thickness. Another role of myofibroblasts is the production and deposition of extracellular matrix proteins, such as collagen, elastin, fibronectin, and tenascin-C, which can induce smooth muscle cell (SMC) proliferation and migration within the media [15, 24, 25]. The medial layer within the vessel contains SMCs, which are regulated under the control of many growth factors and cytokines [26]. These factors influence the phenotype of the SMC inducing proliferation, migration, and contraction, while inhibiting apoptosis. Infiltration of the media with myofibroblasts and hypertrophy of SMCs results in muscularization of normally non-muscular or partially muscular distal arteries augmenting medial thickening. Hypertrophy and excessive proliferation of endothelial cells (EC) contributes to increased intimal thickening and endothelial dysfunction, leading to an increase in vasoconstrictor molecules, such as endothelin-1 (ET-1) [27]. It is suggested that EC dysfunction induces release of elastase, which activates matrix metalloproteinases (MMPs) and in turn tenascin-C, therefore further enhancing SMC proliferation in the medial layer [25, 28].

The progression of pulmonary arterial remodeling is through increased proliferation and resistance to apoptosis of ECs and SMCs. In particular, hyperproliferation of ECs can result in a clustered appearance resulting in the formation of complex plexiform lesions [29], occlusion of the blood vessel, and loss of blood flow to the small distal pulmonary arteries [30]. Although pulmonary arterial remodeling is a relatively well-studied condition, the exact cellular and molecular processes that lead to initiation and progression of PAH are still being investigated and are not completely understood. The process is proposed to be due to changes in transcriptional and signaling mechanisms within all three layers of the vessel wall. Thus, suggesting that controlling gene expression and hence protein expression, will allow modulation of the pathways involved in a cell-specific manner and result in more effective treatments for PAH.

## miRNA biogenesis

**Fig. 2:**
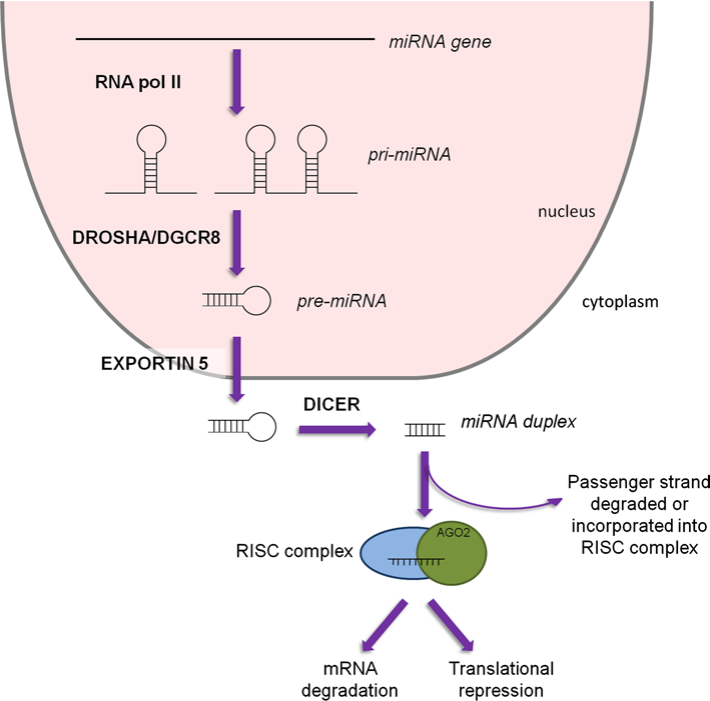
MiRNA biogenesis. Transcription by RNA polymerase II gives rise to the pri-miRNA, which can produce multiple mature miRNAs. Processing by RNase III enzyme Drosha along with cofactor DGCR8 produces the stem-loop pre-miRNA which is exported out of the nucleus by Exportin-5. In the cytoplasm, cleavage by Dicer results in an miRNA duplex, ~22 nucleotides long. The mature miRNA is incorporated into the RNA-induced silencing complex (*RISC*) and targets the 3′-UTR of mRNA. Gene silencing is achieved by either mRNA degradation or translational repression

MiRNAs are small non-coding RNA molecules around 22 nucleotides long that target the 3′-untranslated region (UTR) of mRNA to negatively regulate gene expression [31, 32]. Transcription by RNA polymerase II gives rise to the primary miRNA (pri-miRNA) molecule, which can be over 1 kb in size and can produce several mature miRNAs. This primary structure is processed by the RNase III enzyme Drosha in the presence of cofactor DGCR8 to form a ~60-nucleotide stem loop molecule; this is the precursor miRNA (pre-miRNA). Pre-miRNAs are transported out of the nucleus into the cytoplasm by Exportin-5 where they are cleaved by the endonuclease Dicer into a microRNA duplex. This duplex is then bound to an argonaute protein (Ago2) [33, 34] and the mature miRNA is formed by stabilization of the guide strand (identified by the suffix 5p) while the passenger strand (identified by the suffix 3p) was thought to be degraded, however, there is increasing evidence that the function of 3p-derived miRNAs can be as important as the 5p strand [35, 36] (discussed below). The mature miRNA is incorporated into the RNA-induced silencing complex (RISC) where nucleotides 2–8 of the miRNA (“seed” region) bind to the 3′-UTR of the target mRNA sequence. If the complementarity between mRNA and miRNA is complete, cleavage of the mRNA occurs. However, if complementation is incomplete, inhibition of mRNA translation occurs, leading to gene silencing [32, 37, 38] (Fig. 2). This results in miRNAs potentially regulating a multitude of RNA species [39]. Of the non-coding RNA family, miRNAs are one of the largest families that have a relevant and well-studied function. Little is known about the other members of the non-coding RNA family (e.g., long non-coding RNA, lncRNA) however, recent data implicates these lncRNAs in heart development [40] and consequently these molecules are likely to emerge as important regulators of tissue development within different disease models.

Although the majority of miRNAs follow the canonical biogenesis mentioned above, there are regulatory molecules that aid (or indeed impede) formation of the mature miRNA, thus illustrating how different miRNAs are processed by distinct methods [41]. KH-type splicing regulatory protein (KHSRP) has been found to bind to the terminal stem loop of certain pre-miRNAs (in particular pre-miR-21 and pre-miR-126a) within the nucleus to help recruit Exportin-5 and therefore stimulate miRNA maturation [42]. Similarly, HIV trans-activating response RNA binding protein (TARBP2) can work in concert with Dicer to stabilize the miRNA-Dicer complex as well as ensure efficient recruitment of Ago2 [43]. Conversely, nuclear factor 90 (NF90) and NF45 are found within the nucleus and form a complex that binds to the pri-miRNA, thereby preventing Drosha from cleaving the primary miRNA. This results in reduced levels of pre-miRNA and in turn mature miRNA [44]. It has also been discovered that distinct miRNAs are capable of being processed in a Dicer-independent manner. In particular, miR-451 precursor is a short stem loop sequence and is loaded directly into Ago2 [45, 46]. Of the four argonaute proteins found to be ubiquitously expressed in humans, Ago2 is responsible for the direct cleavage of mRNA when working in concert with RISC. As well as this, Ago2 can also act as an endonuclease and hence cleaves pre-miR-451 independent of Dicer [33, 34, 45, 46].

Until recently, it was thought that the guide strand was always incorporated into the RISC complex and the passenger strand was degraded with no biological role. However, evidence now suggests that both stands of the duplex can have functional roles and both may be important in different signaling cascades [35, 36]. A recent study by Eulalio [35] found that ectopic expression of miR-590-3p and miR-199-3p increased cardiomyocyte proliferation both in vitro and in vivo, illustrating the new emerging role for the 3p strand of the miRNA duplex.

Owing to their pleiotropic vascular effects, miRNAs are thought to function as upstream molecular factors that integratively coordinate pathogenic signaling pathways in PAH. A spectrum of PAH-relevant insults such as hypoxia and inflammation are essential modulators of miRNA expression in vascular cells. Consequently, a number of these dynamically regulated miRNAs are thought to be central regulators in vascular homeostasis essential for thrombosis, metabolism, cellular proliferation, and cell fate. Despite this, only a distinct few miRNAs have been molecularly linked to PAH. Hence, there are undoubtedly a significant number of presently unidentified miRNAs that have an essential role in pulmonary vascular function. Due to the complex molecular pathology of this disease, the combined utilization of bioinformatics and experimental biology may be especially pertinent in deciphering the pervasiveness of miRNA biology in PAH. This review will provide a comprehensive overview concerning miRNA function in pulmonary vascular homeostasis and discuss the contribution of miRNA biology in pulmonary vascular remodeling and disease. We will also speculate on the elucidation of presently unknown miRNAs in PAH and comment on the exciting application of miRNA-based therapies in the treatment of pulmonary vascular disease.

## BMP pathway in pulmonary remodeling

There is a large cohort of data implicating the BMP pathway in the etiology of PAH as heterozygous mutations within the gene encoding bone morphogenetic protein type II receptor (BMPR2) have been found in ~70 % of FPAH [3, 4] and ~26 % of IPAH cases [6]. BMPR2 is a serine/threonine receptor kinase that binds the TGF-β superfamily of ligands. The resulting outcome of BMP signaling is cell and site specific. Under normal conditions, BMP-4 signals via a SMAD-dependent pathway to inhibit proliferation in pulmonary artery SMCs (PASMCs) [47]. TGF-β often antagonizes the effect of BMP signaling and is thought to signal through the activin receptor like kinase (ALK5) and Smad-2 and -3 [48]. The BMP signaling pathway is also controlled by a number of negative regulatory molecules, such as Smad-6/-7, which inhibit phosphorylation of Smad-1 and -5 [49, 50] and Smad ubiquitin regulatory factor 1 (Smurf1) resulting in degradation of Smad-1 and -5 [51]. Chan et al. [52] illustrated that certain proteins interact with the terminal domain of BMPR2 (BMPR2-TD), in particular Tribbles 3 (Trb3). Upon BMP4 stimulation of BMPR2, Trb3 dissociates from the terminal domain of the receptor and binds to Smurf1 inducing degradation of Smurf1 via the ubiquitin–proteasome pathway. Reduction in Smurf1 increases signaling through the BMP-Smad-dependent pathway and promotes the contractile phenotype in vascular SMCs [52].

Many mutations have been discovered within the BMPR2 gene encoding region from patients diagnosed with PAH. However, kindred studies have shown that only ~20 % of people with these mutations go on to develop PAH [5], thus indicating that there may be environmental or additional genetic risk factors involved. PAH patients with a heterozygous mutation in the gene encoding BMPR2 demonstrate lowered levels of BMPR2 protein [53] and PASMCs from these patients have an altered response to the BMP growth factors [54]. Similarly, animal models of PAH show a reduction in BMPR2 protein levels in the lungs [55]. Genetic ablation of BMPR2 proves lethal due to lack of mesoderm [56], while BMPR2^+/−^ mice do not spontaneously develop PAH. However, when BMPR2^+/−^ mice are exposed to a secondary insult (e.g., serotonin), increased pulmonary artery pressures and pulmonary remodeling ensues [57]. This strengthens the idea that BMPR2 mutations predispose patients to develop PAH and that other factors are involved in developing the disease.

## Screening of miRNAs dysregulated in PAH

The first study to report dysregulation of miRNAs during the development of PAH was by Caruso et al. [58]. Using a microarray followed by validation by quantitative polymerase chain reaction, it was found that miR-22, miR-30, and let-7f were downregulated while miR-322 and miR-451 were upregulated in two commonly used rodent models of PAH (exposure to chronic hypoxia and monocrotaline (MCT) insult in the rat) [58]. Another study showed that in human pulmonary arteries from PAH patients, miR-138, miR-367, miR-27b, miR302b, miR-145, and miR-450a were up-regulated while miR-204 was down-regulated compared to control patient pulmonary arteries [59]. This fundamental work highlighted the possible role of specific miRNAs during disease development.

In contrast, others have used a candidate approach to identify miRNAs involved in the remodeling process. MiRNAs which affect signaling pathways involved in the phenotypic changes during remodeling are studied along with their target genes, focusing specifically on the changes in expression during disease initiation and progression [60].

## miR-204

An elegant study by Courboulin et al. [59] was the first to confirm a mechanistic link between miRNAs and cellular pathways affecting pulmonary arterial remodeling. MiR-204 is located within and transcribed from intron 6 of the transient receptor potential melastatin 3 (TRPM3) gene on human chromosome 9. MiR-204 and TRPM3 are transcribed as a single unit [61] however, the effects of miR-204 on signaling pathways involved in PAH do not appear to be mediated via TRPM3 [59]. MiR-204 has been implicated in the development of several tissues (e.g., maintaining epithelial barrier function in eye development [62]) and disease states. The expression of miR-204 has been linked with survival rate in neuroblastoma [63] and miR-204 is found to be down-regulated in tumor tissue from gastric cancer patients [64]. Both studies found that miR-204 directly targets Bcl-2 (B cell lymphoma 2), an inhibitor of apoptosis and with suppressed levels of miR-204, Bcl-2 levels were de-repressed therefore promoting inhibition of apoptosis [63, 64].

With regard to pulmonary remodeling, miR-204 was first observed to be dysregulated in the MCT and hypoxic rat model of PAH with levels of miR-204 down-regulated in the lungs from the disease model [58] as well as in the hypoxic mouse lung [59]. In situ hybridization demonstrated localization of miR-204 within the PASMC compartment and miR-204 expression was reduced in PASMCs from IPAH patients, accompanied by increased levels of proliferation and lower levels of apoptosis compared to control PASMCs. Additionally, treatment of MCT rats with synthetic miR-204 mimic significantly lowered pulmonary artery pressure and medial wall thickness in small pulmonary arteries [59]. It will be important to define, for potential therapeutic purposes, the optimal mimic delivery strategy that leads to effective and safe mimic administration to the pulmonary vascular compartment in vivo.

**Fig. 3:**
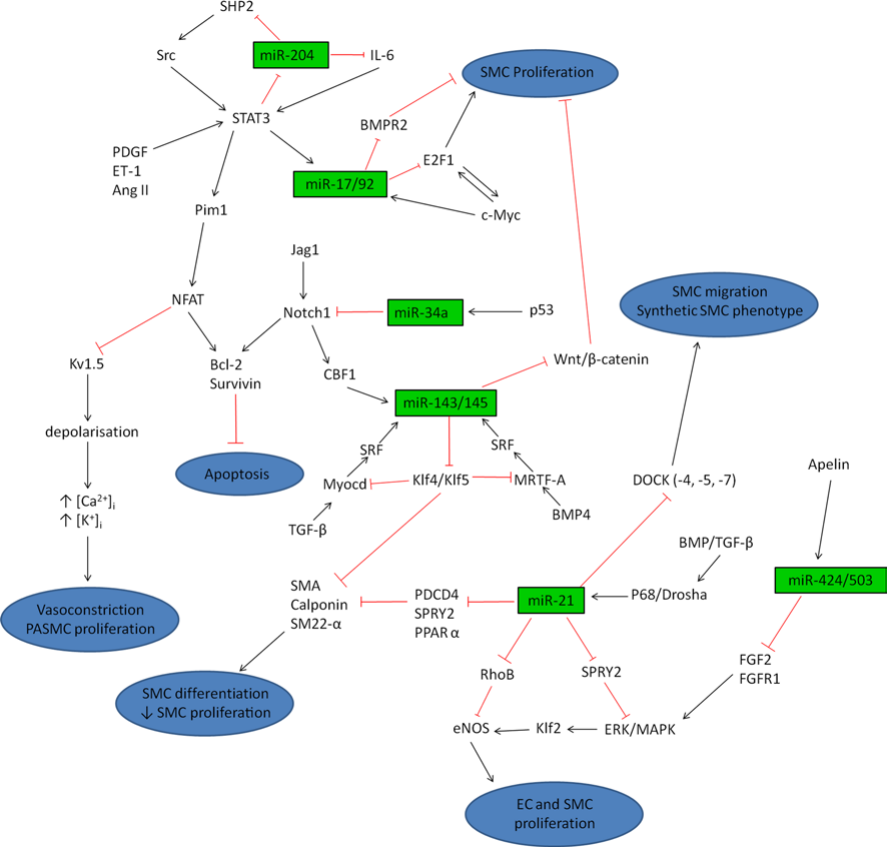
MiRNA regulatory pathways. Summary schematic illustrating the complex pathways controlled by miRNAs. Dysregulation of endothelial cells (*EC*) and smooth muscle cells (*SMC*) can occur in response to stress or injury to the vessel leading to vasoconstriction and pulmonary arterial remodeling

The effect of miR-204 on proliferation and apoptosis is proposed to be via the Src-STAT3-NFAT/BMPR2 pathway (Fig. 3). During the initiation of PAH, circulating vasoconstrictor molecules such as endothelin-1 and PDGF activate STAT3, which can directly bind to miR-204 leading to down-regulation of this miRNA. This causes an increase in miR-204 target gene SHP2, a tyrosine phosphatase involved in growth factor signaling, leading to activation of Src and increasing STAT3 activation [59]. This positive feedback of STAT3 may indicate why the progression of pulmonary arterial remodeling is so severe. STAT3 has been shown to directly activate the serine/threonine protein kinase Pim1 which in turn triggers activation and nuclear translocation of NFATc2. In vivo inhibition of Pim1was able to reverse MCT-induced PAH in rats while Pim1^−/−^ mice were resistant to PAH development [65]. NFATc2 is up-regulated in pulmonary arteries from PAH patients [66] and animal models of PAH have illustrated a down-regulation of voltage-gated potassium channels, in particular Kv1.5 [67]. A key study by Bonnet et al. [66] found that NFATc2 activation decreased the expression of Kv1.5 resulting in PASMC depolarization and opening of the voltage gated calcium channels, hence increasing intracellular levels of potassium ([K^+^]_i_) and calcium ([Ca^2+^]_i_). Increased [Ca^2+^]_i_ promotes further NFATc2 activity and leads to vasoconstriction and PASMC proliferation [68]. NFATc2 also increased anti-apoptotic Bcl-2 leading to mitochondrial hyperpolarization and resistance to apoptosis [66]. Treatment of PAH PASMCs with VIVIT (a direct inhibitor of NFAT) or cyclosporine A (an indirect inhibitor of NFAT), lowered levels of nuclear activated NFATc2, reduced [K^+^]_i_ and [Ca^2+^]_i_ and depolarized PASMC mitochondria to levels comparable to normal PASMCs. Similar results were obtained in vivo using cyclosporine A, with inhibition of NFATc2 reversing MCT-induced PAH by increasing expression of Kv1.5, reducing Bcl-2 levels and promoting mitochondrial-dependent apoptosis. This culminated in reduced right ventricular hypertrophy (RVH) and medial hypertrophy of the small pulmonary arteries [66].

Administration of phenamil, a derivative of the diuretic amiloride, to the hypoxic rat model of PAH caused a reduction in PAH development and pulmonary remodeling. This is thought to be due to phenamil inducing Trb3 therefore increasing signaling through the BMP pathway and resulting in pro-proliferative and anti-apoptotic PASMCs [52, 69]. These findings highlight an essential role of miR-204 in controlling PASMC proliferation and apoptosis via NFAT regulation. There are many molecules involved in the NFAT signaling pathway and modulating either miR-204 or down-stream effectors may provide a target for future therapy of pulmonary remodeling. Consideration has to be given to the method by which these pathways are modulated. Over-expression of miR-204 via synthetic miR mimics would require optimization of mimic delivery to target specifically the pulmonary vasculature therefore limiting off-target effects. Although much work is required to further the present data and before a viable therapeutic agent is produced, the work shows promise.

## miR143/145 cluster

Smooth muscle cells (SMCs), one of the key cellular components of pulmonary vascular remodeling, are able to “phenotypically switch” between the quiescent “contractile” phenotype and the migratory and proliferative “synthetic” phenotype in response to different growth factors to induce repair during vascular injury [70, 71]. Persistent activation of the highly proliferative synthetic state can be detrimental and lead to disease progression [72]. Two of the key miRNAs involved in regulation of the phenotype of SMCs are miR-143 and miR-145. MiR-145 is transcribed bicistronically along with miR-143 from human chromosome 5 [73]. Expression of the miR-143/miR145 cluster is high in cardiomyocytes during heart development, after which expression is exclusively localized within the SMCs [73–75]. Activation of miR143/miR145 is highly regulated through a DNA element called CArG box, contained within the promoter region of the pri-miRNA cluster, which is activated by binding of serum response factor (SRF) and its cofactors myocardin (Myocd) and myocardin-related transcription factors (MRTF) [73]. Growth factors TGF-β and BMP4 have been shown to activate miR-143/miR-145 via distinct mechanisms. TGF-β induces Myocd expression while BMP4 stimulation leads to MRTF-A activation, both pathways resulting in activation of miR-143/miR-145 cluster via the CArG box [70, 76, 77].

With relevance to PAH, miR-143 and miR-145 can potentially target many of the same genes, leading to co-operativity of this cluster in translational regulation. Down-regulation of target genes klf4 and klf5 by activation of miR-143/miR-145 (via TGF-β and BMP4) increases expression of smooth muscle-specific genes such as SMA, calponin, and SM22-α, promoting differentiation and repression of proliferation in vascular SMCs [75, 76, 78, 79]. It has also been shown that klf4 down-regulates Myocd and MRTF-A, two of the critical signaling molecules involved in promoting the contractile phenotype via miR-143/miR-145 activation [76, 78]. Another transcription factor, klf2, has been shown to bind to a promoter region within the miR-143/miR-145 cluster up-regulating expression of this miRNA cluster [80] and stimulating cell–cell communication between ECs and SMCs. Klf2 has been shown to generate the atheroprotective effects in human ECs in response to shear stress, one of the major contributing factors of atherosclerosis [80, 81]. A recent study revealed that over-expression of klf2 in human umbilical vein endothelial cells (HUVECs) led to an up-regulation of miR-143 and miR-145 within the HUVECs but also within the extracellular vesicles released by these cells. These vesicles were then able to control the phenotype of co-cultured SMCs by down-regulating target genes of miR-143/miR-145, therefore promoting the contractile phenotype [80]. Further to this, intravenous injection of extracellular vesicles from ECs known to have elevated Klf2 expression reduced atherosclerotic lesion formation in the ApoE^−/−^ mouse [80].

Recent studies have demonstrated an SRF-independent pathway of miR-143/miR-145 activation [82]. Jag-1 activates translocation of notch intracellular domain, which can then interact with CBF1 regulators. This complex binds to the miR-143/miR-145 promoter region (independent of the CArG box) resulting in up-regulation of SMC contractile genes [82]. These critical studies emphasize the importance of miR-143/miR-145 in regulating and maintaining vascular SMC phenotype under normal conditions.

The SMC phenotypic switch is clearly integral to the pathogenesis of vascular diseases and hence many studies have reported the involvement of miR-143/miR-145 in this setting. Expression of miR-143/miR-145 was significantly reduced in animal models characterized by neointimal formation, including carotid artery ligation, carotid artery balloon injury, transverse aortic constriction (TAC), and ApoE knockout mice [75, 79, 83]. Additionally, over-expression of miR-145 in models of vascular disease has resulted in reduced neo-intimal formation and increased expression of SMC genes, indicative of the contractile phenotype [79, 83]. Use of miR-143/miR-145 knockout mice supports these results with increased neo-intimal formation compared with wild-type mice [74, 79]. Xin et al. [73] found opposing results with miR-143 and miR-145 knockout mice displaying reduced neo-intimal formation in response to carotid artery ligation. However, this result is thought to be due to maladaption of the knockout mice to injury. While these studies highlight the importance and implication of this axis in vascular pathology, they do not directly assess the influence of the miRNAs in PAH.

In the setting of PAH, recent work established that miR-145 was up-regulated in pulmonary arteries from IPAH patients [59], PAH-PASMCs containing a known BMPR2 mutation, and in lung tissue from IPAH and FPAH patients [84]. Expression of miR-145 was noted within the muscular regions of the lesions [84] with greater expression of miR-145 in the concentric lesions compared to plexiform lesions [85]. MiR-145 expression was also significantly increased in the hypoxic mouse model of PAH, both in the lungs and the right ventricle. Furthermore, knockout of miR-145 by both the use of miR-145^−/−^ mice and mice treated with antimiR-145 significantly reduced the systolic right ventricular pressure (RVP) and number of remodeled vessels compared to control hypoxic animals. Target analysis provides evidence that members of the Wnt signaling pathway are targets for miR-145. WIF1, FRZB, and DAB2, which are part of the Wnt signaling family, were increased in hypoxic miR-145^−/−^ mice [84]. In addition to this, other members of the Wnt signaling pathway have previously been shown to regulate PASMC proliferation [86], thus illustrating that the protective remodeling effect observed in miR-145^−/−^ mice may be due to inhibition of Wnt/β-Catenin canonical signaling pathway. Loss of miR-143 did not illustrate the same protective effect as miR-145 [84] however, further studies are required to ascertain the exact role of miR-143 in the pulmonary vasculature and the remodeling process. Taken together, in vitro studies have demonstrated the essential role for miR-143/miR-145 for controlling SMC phenotype. Studies in vivo using rodent models of PAH indicate that specifically knocking down miR-145 expression appears to confer protection against development of PAH. Consequently, miR-145 may be targeted as treatment for pulmonary diseases once more is known about the exact pathways activated within the pulmonary vasculature in response to modulation of miR-145.

## miR-17/92 cluster

As well as regulating the NFAT pathway, miR-204 has also been implicated in regulating the BMPR2 pathway indirectly as miR-204 has been shown to regulate the cytokine interleukin-6 (IL-6). IL-6 has been shown to be involved in PAH development as levels are increased in PAH patients and over-expression of IL-6 induces PAH in mice [87]. In human pulmonary artery endothelial cells (PAEC), IL-6 induces expression of the miR-17/92 cluster via activation of STAT3, which can directly bind to the promoter region of the miR-17/92 cluster (specifically miR-20a). BMPR2 is a target for this miR cluster and accordingly, BMPR2 protein levels are down-regulated in response to IL-6 [88]. Both miR-204 and miR-17/92 cluster are activated through different mechanisms but there are common molecules to both signaling pathways, indicating the importance of miRNAs working in concert with one another to affect cellular phenotype.

MiR-17/92 is a polycistronic cluster located on human chromosome 13, which gives rise to six mature miRNAs; miR17, miR-18a, miR-19a, miR-20a, miR-19b-1, and miR-92-1 [89]. Although all six miRNAs are transcribed together, each miRNA will regulate its own set of targets. Hence, regulation of the miR-17/92 cluster has the potential to control a great deal of target genes. The miR-17/92 cluster was first identified to be involved in tumor development due to up-regulation in tumor cells [90]. O’Donnell et al. [91] demonstrated that activation of the miR-17/92 cluster was via the transcription factor c-Myc. MiR-17-5p and miR-20a down-regulate target gene E2F1, which is involved in control of cell cycle and can induce c-Myc in a feedback loop. The miR-17/92 cluster is highly involved in controlling tumor growth via regulation of proliferation and apoptosis [92] and another cell cycle regulator, cyclin-dependent kinase inhibitor 1A (p21), is also targeted by miR-17. Inhibition of miR-17 in the hypoxic mouse and MCT rat model of PAH caused a reduction in systolic RVP and pulmonary vascular remodeling with an increase in p21 expression [93]. The exact mechanisms through which this miRNA cluster are acting are still relatively unknown, therefore further knowledge must be gained before we can understand the true role of this cluster in the pulmonary vasculature.

## miR-424/503

Recent work has highlighted the role of miR-424/503 in PAH. MiR-424 and miR-503 are located within 250 base pairs of each other on the human X chromosome and are transcribed together [94]. The influence of these miRNAs on pulmonary remodeling has been suggested due to their activation by Apelin. Apelin is highly expressed in pulmonary endothelial cells and expression is decreased in lung ECs and in serum from IPAH and FPAH patients [94, 95]. Apelin^−/−^ mice were shown to develop exaggerated PAH when exposed to hypoxia with obliteration of the small pulmonary arteries, compared to wild-type mice [95]. This finding was proposed to be via Apelin targeting AMP-activated kinase, which in turn activates klf2 to regulate endothelial nitric oxide synthase (eNOS) production [95]. Kim et al. [94] then found that decreased apelin expression in PAECs resulted in up-regulation of fibroblast growth factor 2 (FGF2) and increased proliferation. This study postulates that apelin can target miR-424 and miR-503 in PAECs, which can then directly repress FGF2 and FGFR1. Over-expression of these two miRNAs resulted in down-regulation of FGF2 and FGFR1, reducing phosphorylation of ERK1/2 and inhibition of proliferation [94, 96]. Furthermore, PAECs regulate PASMCs in a paracrine fashion as exposure of PASMCs to media conditioned by PAH PAECs led to hyperproliferation of PASMCs due to increased FGF2 expression [97]. Lentiviral over-expression of miR-424 and miR-503 in the lung of animal models of PAH reduced systolic RVP, RVH, muscularization of small pulmonary arteries and decreased proliferation, highlighting the protective effect of miR-424 and miR-503 in the pulmonary vasculature [94]. From these studies, apelin appears to have a critical function in controlling both PAEC and PASMC phenotype via down-stream signaling.

## The role of miR-21 in PAH

MiR-21 is located on human chromosome 17 within the coding gene for transmembrane protein 49 (TMEM49), however miR-21 has its own promoter region and is therefore transcribed independently [98]. The role of miR-21 in pulmonary arterial remodeling has been investigated in many cell types and in vivo models. As stated before, hypoxia is one of the main triggers for pulmonary remodeling and miR-21 expression is up-regulated in human PASMCs [99], human PAECs [60], and mouse lungs [93, 100] exposed to hypoxia. Increased PASMC proliferation and migration in hypoxic PASMCs or control PASMCs over-expressing miR-21 has been observed, with reversal of phenotype with treatment of antimiR-21 [99, 100]. Down-regulation of miR-21 target genes PDCD4, SPRY2, and PPARα may be responsible for the increased proliferation observed [99, 101] and reduction in PDCD4 has recently been proposed to have anti-apoptotic effects [102].

Bone morphogenetic protein (BMP) signaling also plays a part in modulating miR-21 activation once again in a ligand-specific manner. BMP4 stimulates SMAD-1 and -5, while TGF-β stimulates SMAD-3, to bind to the RNA helicase p68, which is part of the Drosha complex. This complex promotes processing of pri-miR-21 into pre-miR-21, therefore increasing mature-miR-21 expression, which can down-regulate target genes such as PDCD4, resulting in increased smooth muscle contractile gene expression [101]. Upon induction of miR-21 by BMP4, members of the dedicator of cytokinesis (DOCK) 180-related protein family (DOCK-4, -5 and -7) were down-regulated. A reduction in DOCK protein leads to an inhibition of cell migration and promotion of the contractile SMC phenotype [103], thus illustrating another miRNA that can modulate PASMC phenotype via BMP signaling.

Tissues taken from patients with heart failure and animal models of cardiac disease show an up-regulation of miR-21 [104, 105]. However, two separate groups have found opposing results regarding therapeutically regulating miR-21 in the heart. Thum et al. [104] found that the significant up-regulation of miR-21 in cardiac fibroblasts after TAC lead to repression of its target Sprouty homologue 1 (Spry1), resulting in activation of ERK-MAP kinase signaling pathway and increased cell survival. Treatment with antagomiR-21 was able to reverse these changes, leading to a reduction in fibrosis [104]. On the contrary, Patrick et al. [105] found that a reduction in miR-21 did not influence cardiac hypertrophy and fibrosis. In response to various cardiac injuries, including TAC, infusion of AngII and coronary artery ligation, miR-21^−/−^ mice showed comparable phenotypes to the wild-type mice with increased remodeling and fibrosis, with similar results obtained using an LNA-antimiR targeting miR-21 [105]. The differences observed between these two studies may be due to differences in chemistry between the oligonucleotides used for miR-21 knock down. These studies indicate the possible challenges faced when using knock down of a miRNA as a target for prevention and reversal of the diseased state.

Similar to the controversy surrounding miR-21 in cardiac disease, conflicting results have also been obtained from in vivo experiments regarding the role of miR-21 in PAH. Knock down of miR-21 in hypoxic mice using antimiR-21 resulted in a reduction in systolic RVP [93] and lowered muscularization of distal pulmonary arteries, thought to be brought about by a reduction in fibronectin, endothelin-1, α-SMA, Cald1 and SM22-α [100]. On the contrary, Parikh et al. [60] observed an exaggerated PAH phenotype in mice void of miR-21 expression in the pulmonary vasculature after exposure to Sugen-5416 (vascular endothelial growth factor receptor inhibitor) coupled with chronic hypoxia with an increase in systolic RVP and increased pulmonary arterial remodeling shown by increased muscularization in the arterial wall. This amplified response may be due to repression by RhoB, a target of miR-21. RhoB expression induces Rho-kinase activation, which attenuates endothelial nitric oxide synthase, leading to pulmonary vasoconstriction [60]. To support this data, RhoB knockout mice have reduced pulmonary remodeling and RVH when exposed to hypoxia [106].

Conversely, another study has reported that expression of miR-21 was decreased in male rats exposed to MCT with expression unchanged in hypoxic conditions and down-regulation of miR-21 also observed in lung samples from IPAH patients [58]. The variation between the studies mentioned may be due to multiple experimental variations, including differences in species and distinct methods used to modulate miR-21 levels as mentioned above. Another possibility that may lead to differences in findings could be gender. Both males and females are known to have different susceptibility to developing PAH and therefore the miRNA profile may be modulated differently between the sexes. Taken together, these findings highlight the importance of miR-21 in pulmonary remodeling, however, the exact mechanism of action of miR-21 appears to be dependent on various experimental factors.

## Other pathogenic mechanisms linking miRNAs and PAH

Resistance to apoptosis is a major component leading to the development of pulmonary remodeling and recent work has highlighted the role of p53 and miR-34a [107]. MiR-34a is located on human chromosome 1 and pri-miR-34a is a direct target for the tumor suppressor protein p53. Thus, miR-34a is thought to be involved in various cancer pathways [108, 109]. Over-expression of miR-34a leads to a reduction in proliferation and augmented apoptosis [110–112]. A study by Mizuno et al. [107] demonstrated that hypoxia up-regulates p53 and in turn miR-34a. Accordingly, p53 knockout mice display an exaggerated PAH phenotype with increased systolic RVP, RVH, and medial wall thickening in small pulmonary arteries when exposed to hypoxia compared with hypoxic wild-type mice [107]. Notch1 has been verified as a direct target for miR-34a [113], thus over-expression of miR-34a down-regulates Notch1 and its downstream targets Bcl-2 and Survivin [110, 112], both of which are anti-apoptotic. Although the majority of studies reporting the p53/miR-34a/Notch1 pathway have examined the role in cancer, the pathogenesis of cancer and remodeling share several common features, however, further studies will confirm if the same response is initiated in pulmonary artery cells.

MiR-328 is primarily localized within the PASMC and is found to be down-regulated in pulmonary arteries from hypoxic rats and PAH patients. Over-expression of miR-328 in rat PASMC displayed an increase in cellular apoptosis and insulin growth factor 1 receptor (IGF-1R) was identified as a target for miR-328 [114]. This data implicates miR-328 in remodeling by regulating the apoptotic pathway, although the exact molecules and targets involved in the apoptosis response via miR-328 need to be further clarified.

Another miRNA thought to be involved in remodeling is miR-206. MiR-206 is down-regulated in PASMCs from hypoxic mice and hPASMC proliferation and migration is reduced with over-expression of miR-206, along with increases in smooth muscle cell markers therefore promoting the contractile phenotype. Furthermore, hPASMCs over-expressing miR-206 demonstrate increased apoptotic activity, as shown by TUNEL staining and caspase 3 activity [115], indicating that lowered expression of miR-206, as in the case during PAH and hypoxia, promotes excessive proliferation and resistance to apoptosis in PASMCs. The mechanism of action is thought to be due to repression of notch3 by miR-206 as notch3 has been shown to increase SMC growth and prevent SMC apoptosis [116, 117]. Little is known about miR-206 in disease development with previous studies showing its involvement in regulation of breast cancer cell lines [118]. With further in vivo work focusing on up-regulation of miR-206, the pathways and molecules involved in miR-206 modulation can be identified.

## What causes miRNA dysregulation?

The focus of this review has primarily been on the events occurring downstream of miRNA and the ensuing changes in gene regulation and phenotype. However, it is fundamentally important to understand the mechanisms by which these miRNAs are initially regulated during disease. Hypoxia is one of the major rodent models used to study PAH and in addition to this, hypoxia can occur in site-specific regions in response to inflammation or vascular injury. Therefore, one of the key triggers for miRNA dysregulation may be activation of transcription factors, such as hypoxia inducible factors (HIF), which promote regulation of many pathways in response to hypoxic conditions. Mice heterozygous for HIF1α had reduced pulmonary remodeling compared to wild-type mice [119], indicating that HIF1 plays a vital role in controlling vascular remodeling in response to hypoxia. Numerous miRNAs have been identified as being dysregulated in hypoxic conditions in a HIF-dependent manner [120]. Recent studies have found that miR-210 expression is induced by HIF1α in pancreatic cancer cells [121] and endothelial cells [122] therefore providing a possible explanation as to why HIF1α activation can result in gene silencing. As well as inducing miRNA expression, HIF1α can also be targeted by miRNAs to provide a complex and interactive signaling pathway. One such example is miR-155, which has been shown to provide a negative feedback loop involving HIF1α in hypoxic conditions [123].

The cellular response to hypoxia is only one aspect that may promote changes in miRNA expression during disease. Another factor thought to be involved in miRNA dysregulation is inflammation. Inflammation is an important process thought to contribute to PAH phenotype due to the release of cytokines, chemokines, and various growth factors which can result in cellular proliferation, migration and survival. Due to their extensive gene regulatory effects, miRNAs may be involved in the regulation of inflammation, and as a result have an impact on the pathogenesis of PAH. In support of this, Kuhn et al. [124] observed that miR-25 was down-regulated in human airway SMCs in response to pro-inflammatory stimuli including IL-1β, TNF-α, and IFN-γ, while Klf4, a target gene of miR-25, was increased. On the other hand, several miRNAs including miR-155 [125, 126] and miR-146 [126] are induced by various inflammatory mediators. MiR-146a is induced in response to pro-inflammatory stimuli via NF-κB and targets TRAF6 and IRAK1which are molecules involved in NF-κB activation, thus providing a negative feedback loop [126]. Various studies have found an up-regulation of miR-21 expression after exposure to inflammatory stimuli including heat-inactivated bacterium [127], LPS exposure [128], and in a model of allergic airway inflammation [129]. In particular, transcription of pri-miR-21 has been shown to be induced by IL-6, a pro-inflammatory cytokine, in a STAT3-dependent manner [130] and it is believed that initial IL-6 release is due to NF-κB nuclear translocation in response to tissue damage [131]. The fact that miRNAs can be modulated by inflammation as well as target inflammatory mediators adds to the complexity of this regulatory pathway, making it a challenging task to study in a disease model.

Another possible mechanism through which miRNAs are modulated may be through tyrosine kinase pathways. The majority of growth factors (such as PDGF and vascular endothelial growth factor, VEGF) signal through tyrosine kinase receptors that can activate STAT proteins. Recent research has highlighted the function of miRNAs in this pathway as several miRNAs have been shown to be regulated by tyrosine kinase Src and STAT3, in particular miR-204 [59] and miR-17/92 [88] (see individual sections above and Fig. 3). There is strong evidence linking tyrosine kinase dysregulation and signaling dysfunction in disease as the tyrosine kinase inhibitor imatinib was developed as a treatment for chronic myeloid leukemia [132]. Many of the pathologies associated with PAH share similarities with cancer and as a result, imatinib has been the subject of a phase 3 trial as an “add-on” therapy for PAH [133]. Imatinib treatment caused an increase in exercise capacity and improved hemodynamic parameters, however, adverse effects were recorded that limit clinical application in the setting of PAH. Hence, more knowledge is required regarding the exact mechanisms by which tyrosine kinases exert their effect within the pulmonary circulation and miRNAs may provide a crucial link into the downstream effects of this pathway.

One way to characterize the exact processes that are initiating miRNA dysregulation in PAH would be to use a bioinformatics approach, as has been utilized previously [60]. This would allow pathways activated during PAH to be identified and assess the precise mechanisms by which miRNAs are affected. The information gathered from a screening approach would add to the knowledge already known about the downstream effectors of these miRNAs and create a better picture of PAH disease initiation and progression through miRNA pathways.

## miRNAs as biomarkers for disease

The studies presented within this review demonstrate that expression of miRNAs are tissue and cell specific and can be dysregulated during disease. More importantly, many miRNA expression levels correlate with disease severity. This provides a unique signature of miRNAs which are expressed in disease and it is believed that circulating miRNAs can be used as diagnostic biomarkers for early detection of disease. Identifying miRNA profiles in the serum/plasma from certain diseases is ideal, as it is a non-invasive method and miRNAs are highly stable in the blood. It was originally thought that the majority of miRNAs within the circulation were enclosed within exosomes [134, 135] or apoptotic bodies [136], which were released from cells into the circulation, allowing cell to cell communication and miRNA function in cells distinct from where they were synthesized [136]. However, Arroyo et al. [137] used differential centrifugation and size-exclusion chromatography to establish that almost 90 % of circulating miRNAs exist in a protein complex (primarily with Ago2, a key molecule in miRNA biosynthesis). Biomarkers of disease have already been suggested for a number of diseases [138] including heart failure, with miR-423-5p distinguishing diseased state [139] and type II diabetes mellitus characterized with a loss of endothelial miR-126 [140]. There are of course difficulties when analyzing miRNA expression from either serum or plasma, for instance lack of a standard molecule of reference, and further challenges faced in isolating and accurately quantifying circulating miRNAs are discussed in a review by Zampetaki and Mayr [141]. In addition, questions still need to be answered on how the circulating miRNAs are associated with disease initiation and progression and how early these miRNA profiles arise in humans.

## Conclusions

MiRNA research is a relatively recent field and there is still much to discover about the intricate mechanisms involved in the signaling pathways involving miRNAs. It is apparent that miRNAs play a pivotal role in regulating pulmonary arterial remodeling via distinct pathways (Fig. 3). One of the biggest challenges in miRNA research is validating direct targets of specific miRNAs and hence understanding the regulation by which miRNAs exert their effect. Gain- and loss-of-function experiments highlight the beneficial effects of regulating miRNAs and identification of genes involved in the miRNA pathway represent attractive therapeutic targets. The most effective therapies will most likely target the pathways directly involved in controlling pro-proliferation and anti-apoptosis of cells within the vessel wall and reversing these effects. The modulation of miRNAs using either synthetic miRNAs or antimiRs individually or in combination, is highly studied in animal models of PAH, however, there are problems with this treatment. The role of miRNAs throughout the body is broad and the advantage of therapeutically targeting miRNAs is the ability to hit many different pathways involved in disease development and progression. However, this can also be a significant drawback as targeting a miRNA may produce off target effects via modulation of pathways not involved in the diseased state. To overcome this problem, highlighting particular targets of selected miRNAs may prove more successful in reducing disease severity while limiting the effects to pathways involved in PAH. Direct targeting of the miRNA via local delivery to the lung is essential to minimize off-target effects. In addition, directing the miRNA treatment to particular cellular compartments will further reduce unwanted effects. Altogether, the potential to target miRNAs involved in pulmonary remodeling is high and will potentially lead to the generation of novel therapeutic agents.
